# Knockout of *Pi21* by CRISPR/Cas9 and iTRAQ-Based Proteomic Analysis of Mutants Revealed New Insights into *M. oryzae* Resistance in Elite Rice Line

**DOI:** 10.3390/genes11070735

**Published:** 2020-07-02

**Authors:** Gul Nawaz, Babar Usman, Haowen Peng, Neng Zhao, Ruizhi Yuan, Yaoguang Liu, Rongbai Li

**Affiliations:** 1 College of Agriculture, State Key Laboratory for Conservation and Utilization of Subtropical Agro-Bioresources, Guangxi University, Nanning 530004, China; gulnawazmalik@yahoo.com (G.N.); babarusman119@gmail.com (B.U.); phwxx@gxu.edu.cn (H.P.); nengzhao_gxu@163.com (N.Z.); ruizhiyuangxu@126.com (R.Y.); 2 State Key Laboratory for Conservation and Utilization of Subtropical Agricultural Bioresources, South China Agricultural University, Guangzhou 510642, China

**Keywords:** rice, *M. oryzae*, CRISPR/Cas9, *Pi21*, homozygous, resistance, proteomics, iTRAQ

## Abstract

Rice blast (*Magnaporthe oryzae*) is a devastating disease affecting rice production globally. The development of cultivars with host resistance has been proved to be the best strategy for disease management. Several rice-resistance genes (R) have been recognized which induce resistance to blast in rice but R gene-mediated mechanisms resulting in defense response still need to be elucidated. Here, mutant lines generated through CRISPR/Cas9 based targeted mutagenesis to investigate the role of *Pi21* against blast resistance and 17 mutant plants were obtained in T_0_ generation with the mutation rate of 66% including 26% bi-allelic, 22% homozygous, 12% heterozygous, and 3% chimeric and 17 T-DNA-free lines in T_1_ generation. The homozygous mutant lines revealed enhanced resistance to blast without affecting the major agronomic traits. Furthermore, comparative proteome profiling was adopted to study the succeeding proteomic regulations, using iTRAQ-based proteomic analysis. We identified 372 DEPs, among them 149 up and 223 were down-regulated, respectively. GO analysis revealed that the proteins related to response to stimulus, photosynthesis, carbohydrate metabolic process, and small molecule metabolic process were up-regulated. The most of DEPs were involved in metabolic, ribosomal, secondary metabolites biosynthesis, and carbon metabolism pathways. 40S ribosomal protein S15 (P31674), 50S ribosomal protein L4, L5, L6 (Q10NM5, Q9ZST0, Q10L93), 30S ribosomal protein S5, S9 (Q6YU81, Q850W6, Q9XJ28), and succinate dehydrogenase (Q9S827) were hub-proteins. The expression level of genes related to defense mechanism, involved in signaling pathways of jasmonic acid (JA), salicylic acid (SA), and ethylene metabolisms were up-regulated in mutant line after the inoculation of the physiological races of *M. oryzae* as compared to WT. Our results revealed the fundamental value of genome editing and expand knowledge about fungal infection avoidance in rice.

## 1. Introduction

Rice blast is a devastating disease threatening rice production worldwide, causing about 30% estimated yield losses annually [1,2,3]. The most practical approach for managing the blast disease is the development of cultivars with durable resistance by introducing resistance (*R*)-genes and it is the most economical, sustainable, and environment-friendly [1,4]. The immune system of plants has evolved sophisticated defense mechanisms against the various invading pathogens. The plant immune system consists of PAMP-triggered immunity (PTI) and effector-triggered immunity (ETI). PTI response activated by pattern recognition receptors (PRRs), which detects pathogen-associated molecular patterns (PAMPs), is relatively weak and restricts colonization of invading pathogens. PTI is prophesied to confer broad-spectrum and long-lasting resistance without race specificity. Typically, hypersensitivity associated strong response is triggered by the normal defense system. Because of the persistent changes in the pathogenicity of the fungus, generally, the ETI-mediated resistance is highly specific and expected with low durability [5]. Nearly one hundred resistance genes (*R*-genes) have been identified and mapped which induce resistance to *M. oryzae*, and 25 of them have been cloned and characterized until now [6]. The *R*-gene mediated defense mechanism still needs to be elucidated because it is likely to be broken down by highly variable pathogenicity of *M. oryzae*, which makes the rice breeding difficult for durable blast resistance [2].

The comprehensive understanding of defense mechanisms of *R* genes can improve the breeding of rice for durable resistance. *Pi21* is a gene with broad-spectrum resistance that encodes a proline-rich protein that includes a putative heavy metal–binding domain and putative protein-protein interaction motifs. Wild-type (WT) *Pi21* showed plant susceptibility to *M. oryzae* which may support the defense mechanism optimization. The deletion of putative proline-rich motifs for protein–protein interaction causes a loss of function mutation [7]. The *Pi21* knock-out results in non-race specific and durable blast resistance. The rate of hyphal penetration from cell to cell is significantly higher in plants with *Pi21* as compared to the plants having *pi21*, which clearly suggests that the *Pi21* allele negatively regulates the resistance due to its susceptibility factor. The response after the fungus invasion in resistant plants having *pi21* is not as strong or as fast as compared to the response mediated by the *R*-gene. The incomplete resistance or induction of defense mechanisms slowly may lead to a durable resistance [7]. Despite the *pi21* has been identified and cloned already, however, the mechanisms behind resistance against pathogen mediated by the *Pi21* loss of function and the partial and durable resistance is largely unidentified in rice.

As a sequence-specific nuclease (SSN), the CRISPR/Cas9 (clustered regularly interspaced short palindromic repeats/CRISPR-associated protein 9) system has been established as an effective tool for accurate mutagenesis [8,9,10] and is basically derived from Streptococcus pyogenes (SpCas9) adaptive immunity system, in which Cas9 protein is loaded with a synthetic 20 bp long sgRNA (single-guided RNA) and short insertions or deletions (indels) are introduced at the double-strand DNA breaks (DSBs) during the NHEJ (non-homologous end-joining) repair pathway [10]. This system has successfully been applied and targeted mutations were generated at high frequency both in mammalian and plant species, including rat [11], mouse [12,13], human [14,15], rice [16,17,18], Arabidopsis [19,20,21], tobacco [22], maize [23,24], and soybean [25,26]. The CRISPR approach has been successfully employed for the induction of mutation in multiple genes simultaneously [27,28,29] and for target gene activation and repression [30,31]. In early studies, the large fragment deletions in plant cells were rarely achieved but with the advancement in CRISPR/Cas9, numerous reports showed large deletions [17,32].

To perceive the effects of mutations on plant proteome, the proteomics analysis strategy is the most powerful way to explore the diverse changes in complex biological processes and iTRAQ (isobaric tags for relative and absolute quantification)-based proteome analysis is an effective technique for quantitative measurement of protein abundance, regardless of the causes of mutations [33,34]. It has been widely used in plant species to recognize the diverse biological processes including, Arabidopsis [35], rice [36], wheat [37], and maize [38]. A rising number of CRISPR-Cas9 applications necessitate a good understanding and better management of its efficiency and off-targets. Many experimental approaches and prediction tools have been established for achieving these aims [39,40], but it is still required to realize the vastly complex mechanisms underlying cellular signaling processes, which together arrange the response of the cell to genetic mutations and perturbations.

Despite the rapidly emerging efficient technologies, there are presently no iTRAQ-based proteomic studies available to completely understand the phenotype induced by *Pi21* knockout mutants generated by CRISPR/Cas9. In the present study, we used iTRAQ-based proteomic approaches to understand the cellular signaling changes in the *Pi21* mutants compared to the WT. To obtain broad-spectrum resistance, the CRISPR/Cas9 vector containing two target sites of the *Pi21* gene was constructed. In the present report, homozygous and heterozygous *Pi21* mutants were obtained. The homozygous mutant plants with large fragment deletion displayed complete resistance to blast. In summary, our work suggests that the CRISPR/Cas9-based mutagenesis in *Pi21* holds a great potential to improve the resistance of rice against *M. oryzae* and will provide source material for future breeding programs.

## 2. Materials and Methods

### 2.1. Material Used and Experimental Conditions

The seeds of rice variety (VP 1636) were obtained from Prof. Rongbai Li, Wild Rice Group, Rice Research Institute, Guangxi University. The WT and mutant generations were sown during the normal rice-growing season in the experimental field of Guangxi University, and at the Farm of Divisional Headquarters, Sanya Hainan., China. Vector and promoters (Supplementary file 1, Figure S1, S2A–D) were provided by Prof. Yaoguang Liu, South China Agriculture University, Guangzhou, China. The vector pYL CRISPR/Cas9Pubi-H containing hygromycin selectable marker with modified ccdB fragment flanking by two *BsaI* sites, employed for introducing sgRNA cassette (Supplementary file 1, Figure S3A) [28].

### 2.2. Construction of Vector and Rice Transformation

Guide RNAs targeting the second exon of *Pi21* were designed as previously reported [41] using CRISPR-GE (http://sk1.scau.edu.cn/) [42], with higher targeting specificity (Supplementary file 1, Table S2; Figure S3B). The secondary structures of all sgRNA’s were checked by CRISPR-P (http://crispr.hzau.edu.cn/cgi-bin/CRISPR2/CRISPR) (Supplementary file 1, Figure S4A,B). The expression cassette with two sgRNAs was constructed by overlapping PCR. The primers used for constructing sgRNA vectors are given in Supplementary file 1, Table S1. The construction of the expression cassette was accomplished by inserting the oligos into the *BSaI* site of pYLCRISPR/Cas9 (I) [28]. The expression cassette was transformed into *E. coli* DH5α and transformed by the heat shock method as previously explained [43].

Specific primers (SP-L1/SP-R) were used to confirm both target sites in expression cassette (Supplementary file 1, Table S1). The arrangement of sgRNA cassettes driven by U6 promoter-was as follows; LacZ-OsU6a-T1-OsU6b-T2 with corresponding sizes of 629 bp and 515 bp for T_1_ and T_2_ respectively (Supplementary file 1, Figure S5A,B). The positive colonies were detected after DH5α transformation (Supplementary file 1, Figure S5C). The sequence of *Pi21* in WT was amplified, and primers were designed for both targets (Supplementary file 1, Figure S5D). The transformation of embryonic calli of rice seeds was done by *Agrobacterium tumefaciens*-mediated co-cultivation protocol as previously established [44]. In brief, rice grains were sterilized and cultured at 30 °C using a 2N6 medium after de-husking. The scutella were excised from seeds after five days and used for transformation. After three weeks, the proliferated calli were sub-cultured for four days on a fresh 2N6 medium. Actively growing calli were suspended in 2N6L medium and cultured in dark at 25 °C at 125 RPM, and fresh medium was replaced every week. After the four days of the third sub-culture, the cells with the logarithmic phase of growth were selected for transformation. Transformed EHA105 strains were grown on AB medium (containing 50 mgL^−1^ hygromycin and kanamycin) and suspended in AAM medium at a density of 3–5 × 10^9^ cells mL^−1^. The rice tissues were transferred to 2N6-AS medium without rinsing after immersing several minutes in the bacterial suspension. The culture was incubated in dark for three days at 25 °C and after that, the material was rinsed with 250 mgI^−1^ cefotaxime in ddH_2_O and placed on 2N6-CH medium and cultured for three weeks. Proliferated cells were cultured on the N6-7-CH medium and plated on the regeneration medium after ten days and incubated under illumination at 25 °C. The regenerated plants were transferred to the pots and placed in a greenhouse up to maturity.

### 2.3. Genotyping, Screening of Transgene-free Plants, and Off-target Assessment

The genomic DNA extraction from the leaf samples of T_0_ generation was done by established CTAB method [45] and target-specific primers (*Pi21 F&R*) were designed for the amplification of both target sites (Supplementary file 1, Table S1). Sequencing results were decoded and analyzed via an online tool DSDecode (http://skl.scau.edu.cn/dsdecode/) [42]. The T-DNA free plants were screened by using Cas9 detection primers (Cas9-F/Cas9-R), HPT (Hygromycin phosphotransferase), and expression cassette primers (SPL-1/SP-R) (Supplementary file 1, Table S1). Five putative off-target sites were selected against both targets with 3–4 bp mismatches in each site by using CRISPR-GE (http://skl.scau.edu.cn/) (Supplementary file 1, Table S3). The specific primers flanking off-target sites were designed and sequencing was performed (Supplementary file 1, Table S4).

### 2.4. Pathogen Inoculation and Disease Scoring

To assess the resistance level of rice seedlings to *M. oryzae*, the inoculation was done according to a previously established method [46]. Wild-type (WT) and homozygous mutants (GN-3, GN-5, GN-9, and GN-13) seedlings grown in the greenhouse for two weeks. Seedlings were inoculated by the fungal spore spraying (5 × 10^5^ spores/mL) of *M. oryzae* isolates Guy-11 and YN716. The disease scoring performed according to the typical assessment scheme of the IRRI (International Rice Research Institute) and standardized protocol of Mackill and Bonman [47].

### 2.5. Phenotyping of Mutant Lines

The data for agronomic traits such as plant height (PH), flag leaf length (FLL), flag leaf width (FLW), number of panicles (PN), panicle length (PL), number of grains per panicle (GNPP), seed setting rate (SSR), and 1000 grain weight (GW) were recorded for homozygous mutant lines in T1, T2, and T3 generations.

### 2.6. Protein Extraction and Digestion

Total of 100 mg leaf samples of WT (VP-1636) and homozygous mutant line (GN-5) were ground into fine powder in liquid N2 and transferred to pre-cooled acetone (−20 °C) having 65 mM dithiothreitol (DTT) and 10% (v/v) TCA (trichloroacetic acid) was added and mixed thoroughly, precipitated (2 h at −20 °C) and centrifuged (16,000× *g* for 30 min at 4 °C). After careful removal of the supernatant, the pellet was washed thrice with 20 mL pre-cooled acetone. It was kept for half an hour at −20 °C after centrifugation (20,000× *g* for 30 min at 4 °C). The precipitate was vacuum freeze-dried soon after collection and the pellets obtained were mixed with SDT buffer containing 4% SDS, 100 mMTris-HCl, 100 mM DTT, pH 8.0, boiled for 5 min, and then sonicated on an ice bath. After centrifugation, the obtained supernatant filtered over a 0.22 μm Millipore filter. The concentration of proteins was measured by using a BCA kit (Beyo time Institute of Biotechnology, Shanghai, China) and the extracted proteins digested by the FASP procedure [48].

### 2.7. iTRAQ Labeling and Mass Spectrophotometry Analysis

iTRAQ labeling of the digested peptides was conducted by using iTRAQ Reagents 8PLEXKit (Applied Biosystems, Foster City, CA, USA) following the manufacturer’s instructions. The peptides labeled with iTRAQ tags and were combined and vacuum dried by centrifugation. The reversed-phase fractionation of iTRAQ labeled and mixed peptides were done in an 1100 Series HPLC Value System (Agilent Technologies, Santa Clara, CA, USA), reversed-phase nanoflow HPLC (high-performance liquid chromatography), and MS/MS measurements were conducted according to a previously described procedure [49].

### 2.8. Data Analysis

Proteomics data were analyzed by using Proteome Discoverer 2.1 (Thermo Fisher Scientific, Waltham, MA, USA) against (Rice) database with default parameters. The annotations of the DE proteins were conducted using GO database (http://www.geneontology.org/) and proteins were grouped according to their participation in the biological processes (BP), cellular components (CC), and molecular functions (MF). The proteins were further assigned to the KEGG (Kyoto Encyclopedia of Genes and Genomes) database (http://www.genome.jp/kegg/pathway). Enriched GO terms were identified with Fisher’s Exact Test and the Cluster 3.0 software used to perform analysis of DAPs (differentially accumulated proteins). Pathways with FDR-corrected *p*-values ≤ 0.05 considered significant and were displayed by color intensity. STRING was utilized to analyze PPI networks.

### 2.9. RT–qPCR-Based Validation of Proteomic Data

Total RNA was extracted from thirty days old rice plants by using the RNeasy kit (Qiagen). RT-qPCR was performed with the rice Actin gene as an internal control and primers used for qPCR are mentioned in Supplementary file 1, Table S1. The expression levels of genes were measured by 2^-ΔΔCT^ (cycle threshold) method as explained previously [50]. The expression levels of, *Pi21*, *phenylalanine ammonia-lyase gene (OsPAL1)*, *pathogenesis-related protein 1a (OsPR1a)*, *Oryza sativa pathogenesis-related protein 10a (OsPR10a)*, jasmonic acid (JA) synthesis-related gene (*allene oxide synthase* gene; *OsAOS2*), salicylic acid (SA) synthesis and signaling pathway-associated gene (ethylene response transcription factor; *OsRAP2.6*), and plant defense-related gene *Osβ-1, 3-glucanase* (*Osg1*) were also analyzed before inoculation and 12 h and 24 h after inoculation.

### 2.10. Statistical Analysis

The agronomic data were analyzed using SPSS 16.0 Statistical Software Program. GraphPad Prism (version 7.0, GraphPad Software Inc., San Diego, CA, USA) was used to develop the graphs. The data were analyzed by a two-tailed Student’s t-test and presented as the mean ± SD. The data were considered statistically significant at a *p*-value of <0.05.

## 3. Results

### 3.1. Construction of Vector

The overlapping PCR was performed for the amplification and confirmation of sgRNA expression cassette and TaKaRa MiniBEST Purification Kit Ver.4.0 was used to purify the PCR product. Specific SP-L1/SP-R primers (Table S1) were used for Sanger sequencing. The sequencing results confirmed the successful construction of the CRISPR/Cas9 binary vector having both the sgRNAs sequences (Supplementary file 1, Figure S5E).

### 3.2. Mutation Frequency, Off-Target Effects, and Segregation of Mutants

Hygromycin phosphotransferase primers (HPT-F/R) were used to select mutant plants (Supplementary file 1, Table S1), and the amplified product was confirmed in 25 regenerated mutant lines by gel electrophoresis (Supplementary file 1, Figure S5F). From 145 *A. tumefaciens* inoculated calli we obtained a total of 25 rice seedlings with a transformation efficiency of 17.24%. The results showed that for Target1 there were 6 homozygous (24%), 9 heterozygous with 2 mono and 7 biallelic (36%), 2 chimeric (8%), and 8 (32%) were WT plants; while, for the Target2, there were 5 homozygous (20%), 10 heterozygous with 4 mono and 6 biallelic (40%), 1 chimeric (4%) and 9 (36%) were WT plants. We observed some large fragment deletion of 51 bp in homozygous line GN-5 and biallelic line GN-23 showed 2 and 50 bp deletion in first and second allele respectively. A high mutation rate in both the targets of *Pi21* was observed with a total mutation frequency of 66% (68% for T1 and 64% for T2) with heterozygous mutations were more frequent (Table 1; Supplementary file 1, Table S5).

All the T_1_ plants obtained from T_0_ homozygous plants of GN-3, GN-5, GN-9, and GN-13 showed homozygosity for the same mutations, which indicated the stable transmission of mutations following the Mendelian law. The Biallelic and heterozygous T_1_ plants of GN-19, GN-15, and GN-25 segregated according to Mendelian inheritance (1:2:1) by the chi-square test (Supplementary file 1, Table S6). The target regions of *Pi21* were amplified from the genomic DNA of T_0_ transgenic plants separately and the sequencing chromatograms with overlapping traces were decoded. The DNA of 35 mutant plants was amplified by using specific primers for five selected putative off-target sites of each target showing 3 to 4 mismatching bases. The results showed that there were no secondary off-target mutations detected (Supplementary file 1, Table S3)

### 3.3. T-DNA-Free Plants in T_1_ Generation

**Figure 1:**
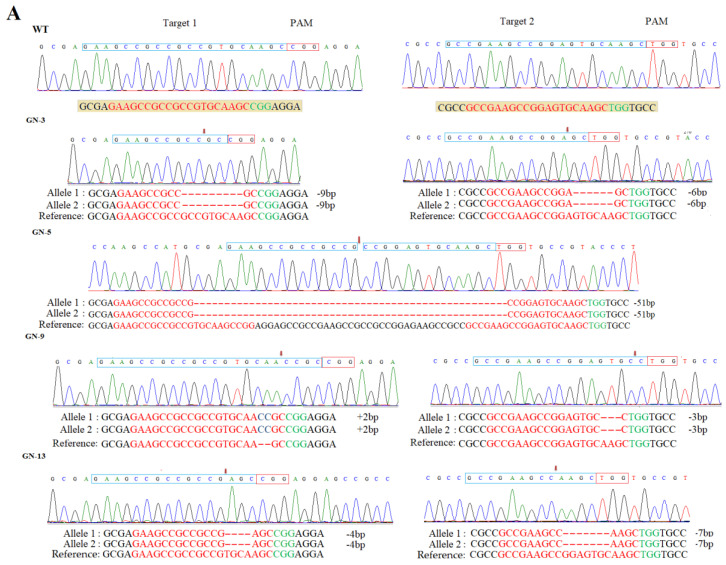

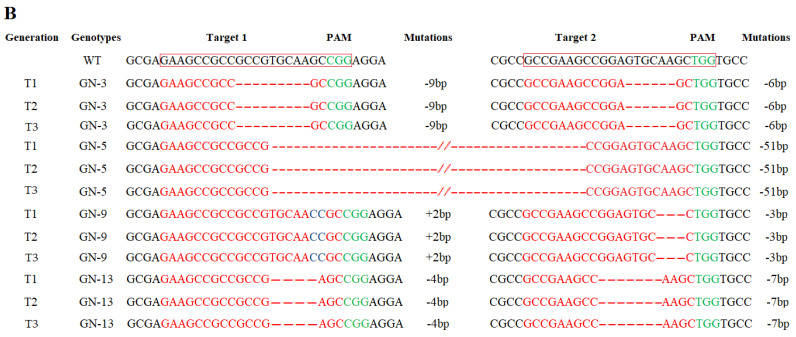
Detection of mutations induced by CRISPR/Cas9; (**A**) chromatograms showing the sequencing results for both the target sites in mutant lines; (**B**) inheritance of mutations in T_1_ and T_2_ and T_3_ generations. Red hyphens and letters represent the deletions and blue letters represent insertions, respectively while the PAM sequence is highlighted in green.

Screening of T-DNA free plants was carried out by using Cas9 detection primers (Cas9-F/Cas9-R), HPT (Hygromycin phosphotransferase), and expression cassette primers (SPL-1/SP-R) (Supplementary file 1, Table S1). We selected four homozygous, 2 biallelic, and one heterozygous line and tested 50 plants of each to confirm the presence or absence of T-DNA. All the lines resulted in the different numbers of transgene-free plants (Supplementary file 1, Table S6). The homozygous line GN-5 showed 17 T-DNA free plants (Supplementary file 1, Figure S5G). Furthermore, the four T-DNA free homozygous lines (GN-3, GN-5, GN-9, and GN-13) were investigated to assess the stability of mutations. GN-3 showed homozygous mutations with 9 bp and 6 bp deletions on the first and second target sites, respectively. Homozygous mutant GN-5 showed 51 bp and 0 bp deletions on first and second target sites, and a 51 bp large fragment deletion between two targets respectively. The homozygous mutant line GN-9 resulted in 2 bp insertion and 3 bp deletion on the first and second target sites. Finally, GN-13 exhibited 4 bp and 7 bp deletions on the first and second target sites, respectively (Figure 1A). The results obtained through Sanger sequencing of T_1_, T_2_, and T_3_ generations confirmed that the resulted mutations were inheritable (Figure 1B).

### 3.4. Resistance to Pathogen in Pi21 Mutants

**Figure 2:**
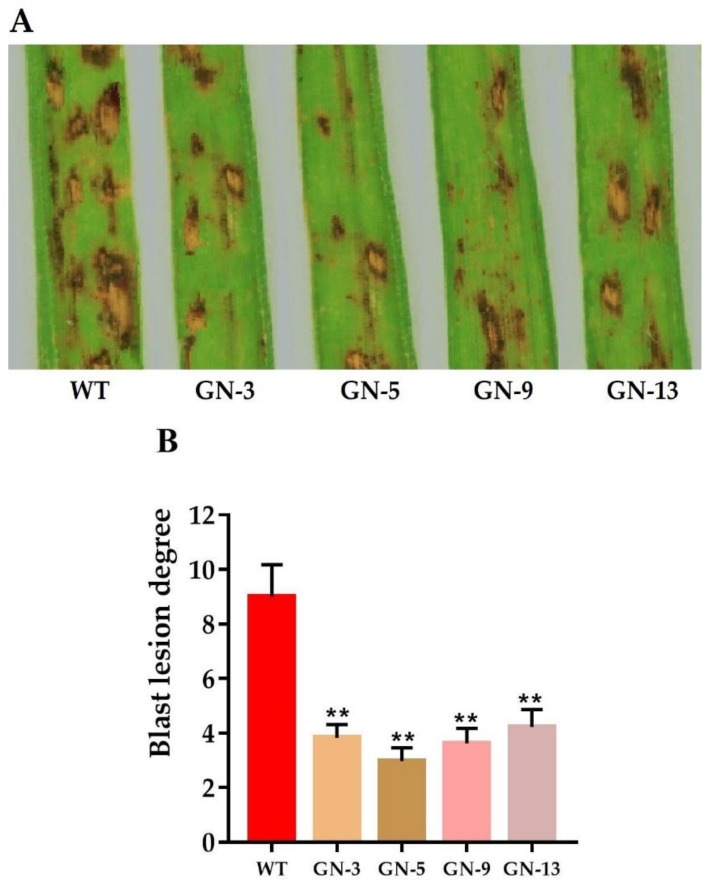
Phenotypic appearance of wild-type and mutant lines inoculated with *M. oryzae* (**A**) after 5 dpi and (**B**) Blast disease scoring in WT and mutant plants “**” denotes the significant difference, student’s *t*-test, *p* ≤ 0.01, *n* = 10.

To investigate the resistance of homozygous mutant lines to *M. oryzae*, we selected four mutant lines GN-3 with 9 bp and 6 bp deletions, GN-5 with 51 bp and 0 bp deletions, GN-9 with 2 bp insertion and 3 bp deletion, and GN-13 with 4 bp and 7 bp deletions on first and second target sites, respectively. WT and homozygous T_2_ mutants were inoculated with *M. oryzae* isolates GUY-11 and YN716 to evaluate the resistance at the seedlings stage. At 5 dpi (days post-inoculation), WT plants were more severely infected, with obvious lesions spreading, while the lesions on the homozygous mutant lines were significantly smaller than those of the WT (Figure 2A). The plants with resistance to the blast were scored as 0–3, while plants scored as 4–9 were considered susceptible. The WT showed the blast lesion degree score of 9, while mutant line GN-3, GN-5, GN-9, and GN-13 showed the blast lesion degree of 3.83, 2.97, 3.62, and 4.22 respectively. The homozygous line GN-5 with large fragment deletion of 51 bp showed the highest resistance against *M. oryzae* among all. These results indicate that *Pi21* loss-of-function mutants developed by CRISPR/Cas9 system had enhanced resistance to rice blast at the seedling stage compared to wild type (Figure 2B).

### 3.5. Agronomic Performance Assessment of Wild Type and Mutant Lines

To investigate whether the mutants generated by the targeted mutagenesis of the CRISPR/Cas9 system can cause changes in the agronomic traits of the mutant plants, we recorded data for the main agronomic traits like PH, FLL, FLW, PN PL, GNPP, SSR, and GW (g) for WT and the four homozygous mutant lines GN-3, GN-5, GN-9, and GN-13 in T1, T2, and T3 generations respectively. The agronomic traits of WT and homozygous mutants remained the same indicating that the CRISPR/Cas9 system targeting the *Pi21* gene has no significant effect on the major agronomic traits (Table 2).

### 3.6. Proteins Extraction and Quantification

**Figure 3:**
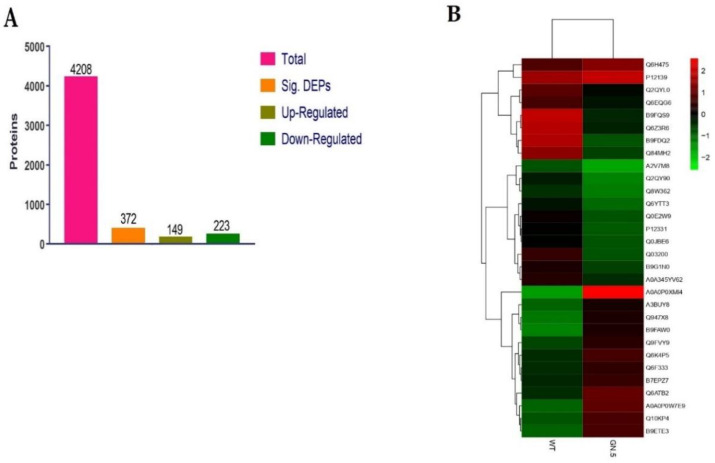
Identified proteins and heatmap of wild type and CRISPR mutant line (GN-5). (**A**) The number of the total, and differentially expressed proteins (up and down-regulated). (**B**) Proteins with higher expression difference among WT and mutant. Red color denotes higher while the green color is representing the lower level of expression.

A total of 543,998 spectra, 40,859 peptides, 6597 proteins, 4208 protein groups, were found in proteomic analysis. The deviation between the measured molecular weight of the peptide precursor and the theoretical molecular weight is shown in (Supplementary file 1, Figure S6a). The concentrated peak around 0 means the mass deviation is small. Proteins with 30–40 kDa accounted for more protein numbers in the protein molecular weight distribution (Supplementary file 1, Figure S6b). Total of 6597 proteins were identified by arranging the data, and finally, 4208 proteins were obtained after adjusting 10 filter cutoff. The comparison of WT (VP-1636) and its CRISPR/Cas9 mutant GN-5 showed 372 differentially expressed proteins (DEPs), 223 down-regulated, and 149 upregulated proteins according to the log2 fold change ≥ 1.2 and *p*-value < 0.05 (Figure 3A). Some DEPs having a higher association with photosynthesis, response to stimulus, and biotic stress tolerance are considered as important proteins (Table 3). The complete information of all the identified proteins is mentioned in Supplementary file 2. The A0A0P0XMI4 (γ-tubulin complex component), A0A0P0W7E9 (Glycosyltransferase), and B9ETE3 (Glutaredoxin domain-containing protein) exhibited higher expression in GN-5 and lower expression level in WT while B9FDQ2 (Uncharacterized protein), B9FQS9 (Uncharacterized protein), and Q6Z3R6 (Os08g0506700) were highly expressed in WT, while exhibited a lower expression level in GN-5 (Figure 3B).

### 3.7. Functional Networks of DEPs

**Figure 4:**
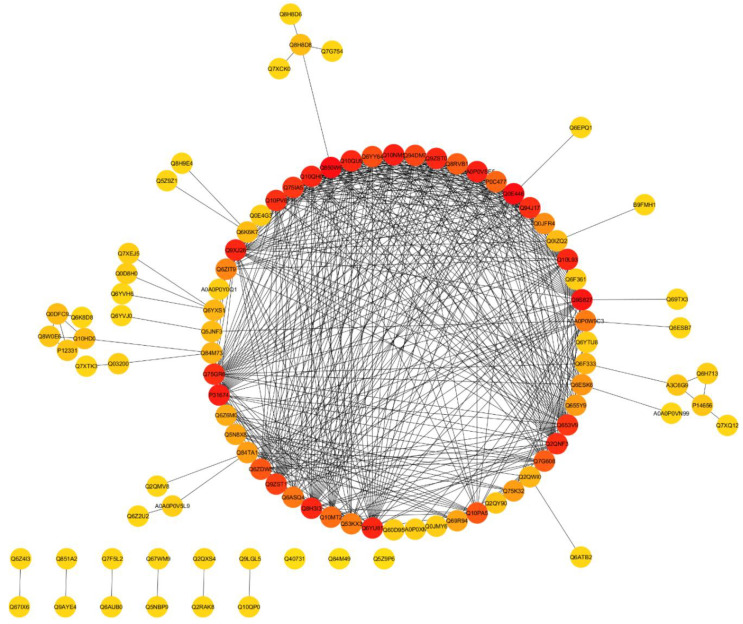
STRING predicted protein to protein interaction (PPI) of differentially expressed proteins (DEPs) between wild type and mutant lines (GN-5). Nodes with redder color show a higher co-expression level.

STRING database was used to retrieve the protein interactions to reveal a putative protein association network. The maximum confidence (score) cutoff was adjusted to 0.60 with 20 maximum additional interactions. The proteins with a higher interactor score of ≥5 were selected. The results showed that the higher co-expression was found between ribosomal protein S5 (Q850W6, Q6YU81), 50S ribosomal protein L4, L5, L6, L13, L15, L17 (Q10NM5, Q9ZST0, Q10L93, Q94J17, Q10PV6, Q75GR6, succinate dehydrogenase (Q9S827), 40S ribosomal protein S15, SA (P31674, Q10QU9, Q8H3I3), 30S ribosomal protein S9, S10, S13 (Q9XJ28, Q10QH0, Q75IA5, and ribonucleoprotein (Q10PA5, Q7G60) (Figure 4).

### 3.8. Gene Ontology (GO) Annotation of DEPs

**Figure 5:**
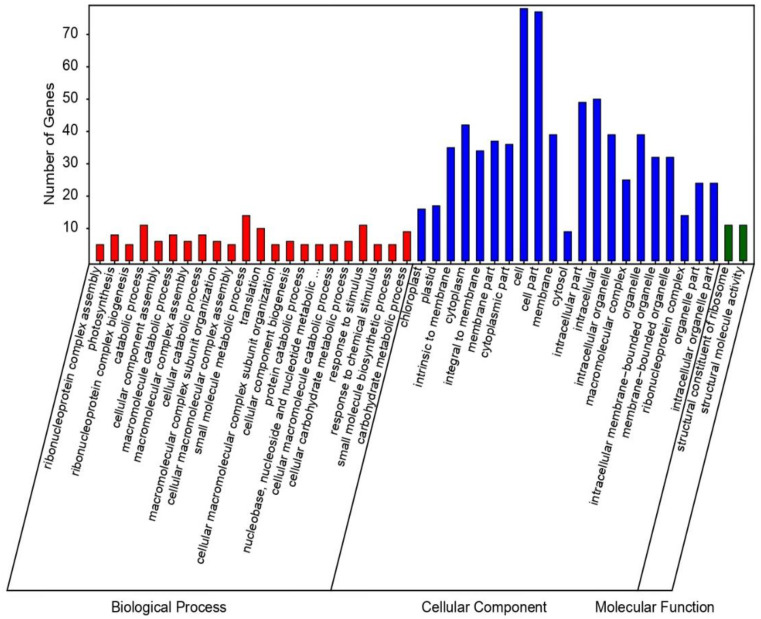
Gene ontology (GO) pathways of up-regulated proteins of WT and mutant line GN-5.

Most of the up-regulated DEPs related to biological processes (BP) were associated with photosynthesis, response to the stimulus, small molecule metabolic process, translation, carbohydrate metabolism, and catabolic process. Proteins conferring cellular component (CC) associated with the cell, cell part, intracellular part, and intracellular process were significantly presenting. Finally, from the molecular function (MF) perspective DEPs involved in structural molecule activity and structural constituent of ribosome, were up-regulated (Figure 5). GO annotations for biological process (BP) exhibited that the down-regulated DEPs mostly participated in the metabolic process, derivative metabolic process, catabolic process, and cellular ketone metabolic process. Proteins conferring cellular component (CC) associated with the cell part, cell, membrane, and membrane part were down-regulated significantly. For molecular function (MF) the down-regulated DE proteins were mainly enriched in oxidoreductase activity, tetrapyrrole binding, iron ion binding, and heme-binding (Supplementary file 1, Figure S7A).

### 3.9. Pathway Enrichment Analysis

**Figure 6:**
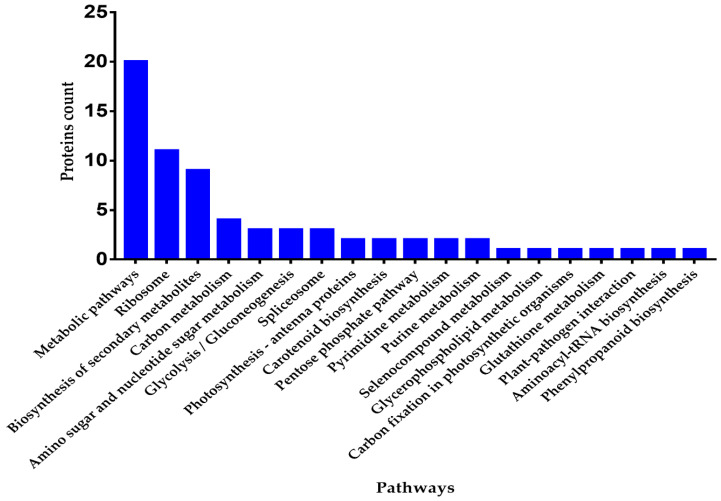
KEGG pathway enrichment histogram for up-regulated proteins (*p*-value ≤ 0.05).

KEGG enrichment analysis of up-regulated DEPs evidenced that the pathways related to metabolic process, ribosome, secondary metabolites biosynthesis, carbon metabolism, glycolysis/gluconeogenesis, spliceosome, amino sugar, and nucleotide sugar metabolism, photosynthesis - antenna proteins, carotenoid biosynthesis, plant-pathogen interaction, aminoacyl-tRNA biosynthesis, and phenylpropanoid biosynthesis (Figure 6). While the down-regulated DEPs showed significant enrichment in glyoxylate and dicarboxylate metabolism, metabolic pathways, processing of proteins in endoplasmic reticulum, secondary metabolites biosynthesis, biosynthesis of phenylpropanoid, mRNA surveillance pathway and alanine, aspartate, and glutamate metabolism pathways (Supplementary file 1, Figure S7B).

### 3.10. Identification of Hub-Proteins

**Figure 7:**
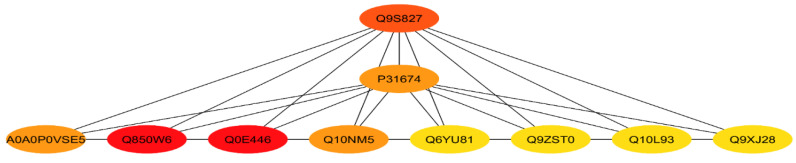
The hub-proteins selected from the total DEPs of WT and GN-5. Every node represents proteins and the edges indicate connections between the nodes, the red color denotes the higher co-expression.

The analysis for top-ten hub proteins revealed that the 40S ribosomal protein S15 (P31674), 50S ribosomal protein L4, L5, L6 (Q10NM5, Q9ZST0, Q10L93), 30S ribosomal protein S5, S9 (Q6YU81, Q850W6, Q9XJ28), and succinate dehydrogenase (Q9S827) were hub proteins (Figure 7).

### 3.11. RT-qPCR-Based Analysis of CRISPR Mutants

**Figure 8:**
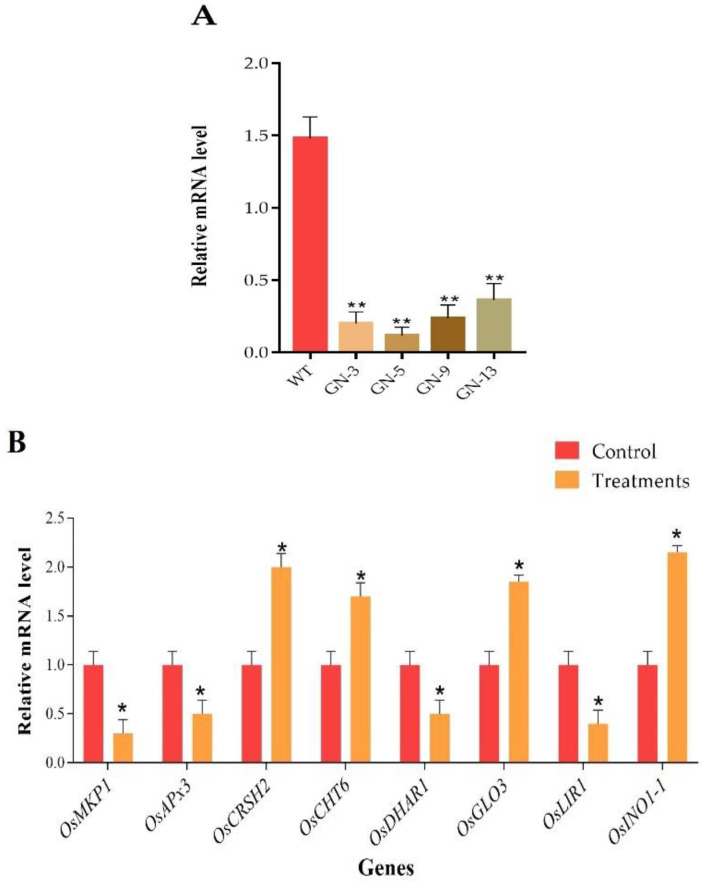

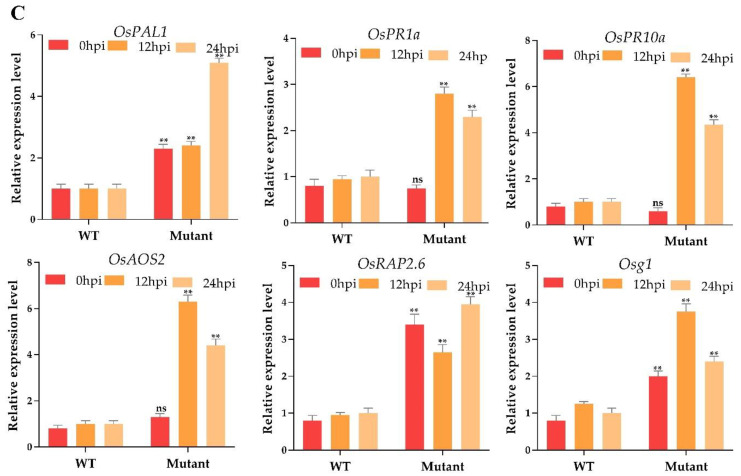
RT-qPCR results for target genes and proteomic data. (**A**) *Pi21* and (**B**) eight selected DEPs responsive genes. (**C**) Relative expression of defense-response genes in WT and the homozygous mutant line inoculated with *M. coryza*. hpi; hours post-inoculation. “*” denotes the significant difference, *p* ≤ 0.05 and “**” denotes the significant difference, student’s *t*-test, *p* ≤ 0.01, *n* = 3.

Total RNA extracted from thirty-days-old rice plants and RT-qPCR was performed to assess the expression level of *Pi21* in mutant lines. The RT-qPCR results unveiled that the expression of *Pi21* was significantly suppressed in mutant lines as compared to WT (Figure 8A). For the verification of differential expression levels of some genes, eight genes were selected for DEPs based on publicly available microarray database. In total, four key genes encoding downregulated proteins including serine carboxypeptidase 3 (*OsACBP3*), MAP kinase phosphatase (*OsMKP1*), probable L-ascorbate peroxidase 3, peroxisomal (*OsAPX3*), probable glutathione S-transferase (*OsDHAR1*), and light-regulated protein (*OsLIR1*), and four genes encoding up-regulated proteins including, probable GTP diphosphokinase CRSH2, chloroplastic (*OsCRSH2*), pathogenesis-related (PR)-3 chitinase 6 (*OsCHT6*), peroxisomal (S)-2-hydroxy-acid oxidase (Os*GLO3*), and inositol-3-phosphate synthase 1 (*OsRINO1*) were selected. The results of RT-qPCR validated the proteomic data findings (Figure 8B). The expression level of the defense-related genes in WT and the homozygous mutant line is shown in Figure 8C. The expression levels of *OsPAL1*, *OsPR1a*, *OsPR10a*, *OsAOS2*, *OsRAP2,* and *Osg1* in mutant line were significantly up-regulated compared with WT after 12 h and 24 h of inoculation while the expression of defense-related genes (*OSPAL1* and *Osg1*) was greater than that of the WT before the inoculation, and the expression level was continuously up-regulated after 12 h and 24 h of inoculation.

## 4. Discussion

Genome editing using SSNs to create specific knockouts have been extensively used in important crops. However, the development of novel disease-resistant genotypes remained limited. In previous studies, the sucrose efflux transporter gene *OsSWEET14* was targeted by TALENs and disease-resistant lines were successfully attained with normal phenotype [51]. Moreover, the stable wheat mutant lines showing durable resistance to powdery mildew were developed by knock-out of three *mildew-resistance locus (MLO)* through TALENs [52]. The transgene-free resistant lines were developed through CRISPR/Cas9-directed editing of *Os8N3* against *Xanthomonas oryzae pv. oryzae* with normal phenotype in rice [53]. In this study, we obtained homozygous *Pi21* mutant plants without any T-DNA integration. The inoculation experiments proved that the mutants were highly resistant to *M. oryzae*, thus can be useful for future breeding programs. The mutant lines were obtained without any off-target mutations, the previously reported work also suggests that the Cas9 rarely tempts off-target mutations in rice [54,55]. The transgene-free plants were screened in a frequency ranging between 18–34% in different homozygous, heterozygous, and biallelic lines with stable transmission of mutations (Supplementary file 1, Table S6). The homozygous mutant lines revealed improved blast resistance with the highest degree of resistance against *M. oryzae*, while the WT plants were completely dead (Figure 2A).

Here we performed the comparative iTRAQ-based proteomic analysis of WT and homozygous mutant line GN-5 to elucidate the changes induced by gene editing at the proteome level. A total of 372 DEPs were identified containing 149 up and 223 down-regulated proteins respectively. The KEGG analysis showed that the metabolic pathways, ribosome, secondary metabolites biosynthesis, carbon metabolism, metabolism of amino and nucleotide sugar, gluconeogenesis/glycolysis, spliceosome, photosynthesis-antenna proteins, carotenoid biosynthesis, and plant-pathogen interaction were highly enriched pathways. Metabolic processes play a significant role within a variety of plant species, the differentially expressed protein Q6ATB2 (probable GTP diphosphokinase CRSH2, chloroplastic) related to this process was up-regulated in mutant line. CRSH2 possesses calcium-dependent ppGpp (guanosine 3′-diphosphate 5′-diphosphate) synthetase activity, it may be involved in response to environmental stress, physical injury, and pathogen attack [56].

In Arabidopsis, the overexpression of chloroplastic NADPH-dependent thioredoxin reductase enhances leaf growth while the cytosolic NTRA confers stress tolerance in plants [57,58]. Secondary metabolites like flavonoids and phenolic acid are ubiquitous in plants and described as markers of abiotic and biotic stress tolerance [59]. In plants, photosynthetic assimilation of carbon is reduced by the carbon metabolism which enhances the ROS production through cellular homeostasis disruption [60]. Previous reports revealed important immune modules that are critical for rice defense responses against blast disease, such as the improved ability of ROS production [5].

GO analysis revealed that most of the DEPs participated in photosynthesis, response to stimulus, metabolic process of small molecules and carbohydrates, translation, and catabolic processes. The photosystem is badly affected by the damages caused by attacking pathogens and it may lose its function, if not timely repaired [61]. Photosynthesis-related proteins (E9KIQ4, Q8W0E6, Q6AVG2, Q2QTK0, Q67WJ2, Q10HD0, Q7FAS1, B9FFA5) were found to be upregulated in homozygous mutant line. Proteins related to response to stimulus (A0A0P0W9C3, Q6K8R2) were up-regulated in homozygous mutants, mainly involved in chitinase activity and defense response to the fungus in rice, as pathogenesis-related protein-3 chitinase 6 play an essential role in plant defense [62], Q6K4P5 (diacylglycerol kinase) play an imperative role in PA signaling of plants [63] evolving as the unique second messengers in the defense system of plants [64] overexpression of *OsBIDKI* showed elevated resistance to disease in tobacco transgenic plants [65]. DEPs belonging to the peroxidases, which create a blockade to pathogen entry [66], were upregulated (Q2R351, A3BUY8, Q75IS1,) in GN-5 compared to WT, suggesting a possible resistance mechanism of *Pi21* mutants to the rice blast.

In hub-proteins network analysis, we found some essential proteins related to metabolic processes and response to stimuli, including ribosomal protein S5 containing protein, expressed (RPS5), 50S ribosomal protein L5, chloroplastic (RPL5), 40S ribosomal protein S15 (RPS15), 50S ribosomal protein L6, putative, expressed (Os03g0356300 protein), 30S ribosomal protein S9, chloroplast, putative, expressed (RPS9) and Succinate dehydrogenase (SDH2-1), protein were top co-expressed. The ribosomal protein S5 containing protein (RPS5) play a prominent role in photosynthesis in plants [67]. Furthermore, the ribosomal proteins are mainly involved in RNA-binding and translation activities and have a key role in the plant immune system, development, and growth [68,69]. Succinate dehydrogenase (SDH) regulates the plant development and salicylic acid (SA)-based signaling, plays an important role in stress response [70,71,72]. The results of proteomic data were validated by RT-qPCR for eight selected genes (*OsMKP1, OsAPx3, OsCRSH2, OsCHT6, OsDHAR1, OsGLO3, OsLIR1, OsINO1-1*). According to the expectations, the results were highly correlated with proteomic findings (Figure 8B). The expression level of SA, JA, and ethylene signaling pathways-related genes (*OsPAL1*, *OsPR1a*, *OsPR10a*, *OsAOS2*, *OsRAP2,* and *Osg1*) was higher in mutant plants as compared to wildtype. Ethylene, JA, and SA play a central role in plants against many types of stresses including abiotic and biotic stresses [73]. Previous studies have reported that the *Osg1* gene encodes a glucanase that degrades the fungal cell wall and found in the cell walls of plant tissues and plant fungal pathogens [74]. *OsRAP2.6* gene is an ethylene-responsive transcription factor that interacts with *RACK1* to participate in innate immune responses in rice plants [75]. 

These results provide an exceptional molecular mechanism underlying loss of function mutations in *Pi21*. Previously so much work has been done on rice blast resistance but, the generation of resistant mutant lines through CRISPR/Cas9-directed mutagenesis of *Pi21* and iTRAQ-based proteomics analysis to unravel changes at the proteomic level is a novel approach. The homozygous mutant lines will provide a source material for rice breeding programs against *M. oryzae* and will present a valuable channel for studying the mechanism of *Pi21*-mediated defense in rice.

## 5. Conclusions

The generation of T-DNA free homozygous mutant lines with the stable inheritance of mutations without any off-target effects are of great value. In this study, we have developed the transgene-free homozygous mutant lines having no off-targets without affecting the major agronomic traits. The comparative proteome profiling of the mutant lines and its WT was performed for the further elucidations of gene editing effects on the proteome profiles of *Pi21* mutants, the comparison of WT (VP-1636), and its CRISPR/Cas9 mutant GN-5 showed 372 differentially expressed proteins (DEPs), 223 down-regulated and 149 upregulated proteins and DEPs related to abiotic and biotic stress resistance were investigated. We have generated the mutant lines with a different frequency of allelic mutations and found that CRISPR/Cas9-guided targeted mutagenesis of *Pi21* resulted in improved resistance to rice blast in elite rice line. The results provided an exceptional molecular mechanism underlying the *Pi21* loss of function mutations. The homozygous mutant lines will provide source material for rice blast resistance improvement.

## Figures and Tables

**Table 1:** Mutation rates of both targets in T_0_ generation.

Mutation Type							
Targets		Bi-Allelic	Homozygous	Heterozygous	Chimeric	WT	Total
*Pi21*T1	NOP	7	6	2	2	8	25
	MR	28.00	24.00	8.00	8.00		68.00
*Pi21*T2	NOP	6	5	4	1	9	25
	MR	24.00	20.00	16.00	4.00		64.00
						Mean MR	66.00%

NOP: number of plants; MR: mutation rate (%); WT: wild type.

**Table 2:** Performance of wild type and homozygous mutant plants for main agronomic traits in T_1_, T_2_, and T_3_ generations.

Generation	Line	PH (cm)	FLL (cm)	FLW (cm)	PN	PL (cm)	GNPP	SSR (%)	GWT (g)
	WT	105.8 ± 4.3	37.36 ± 1.4	1.63 ± 0.2	10.81 ± 0.7	21.36 ± 1.8	155 ± 12	82.12 ± 5.5	29.34 ± 1.1
	GN-3	102.5 ± 2.3 ^ns^	36.55 ± 1.6 ^ns^	1.51 ± 0.3 ^ns^	10.44 ± 0.4 ^ns^	21.84 ± 1.9 ^ns^	158 ± 10 ^ns^	83.10 ± 4.1 ^ns^	28.63 ± 1.6 ^ns^
T_1_	GN-5	106.6 ± 3.4 ^ns^	35.23 ± 1.5 ^ns^	1.67 ± 0.2 ^ns^	9.98 ± 0.6 ^ns^	20.70 ± 2.2 ^ns^	159 ± 11 ^ns^	85.33 ± 4.5 ^ns^	29.93 ± 1.1 ^ns^
	GN-9	107.4 ± 3.8 ^ns^	35.88 ± 1.3 ^ns^	1.64 ± 0.3 ^ns^	10.12 ± 0.5 ^ns^	21.30 ± 1.7 ^ns^	157 ± 12 ^ns^	82.03 ± 3.7 ^ns^	28.43 ± 1.5 ^ns^
	GN-13	108.1 ± 3.6 ^ns^	36.28 ± 1.6 ^ns^	1.59 ± 0.4 ^ns^	10.29 ± 0.3 ^ns^	21.88 ± 1.5 ^ns^	156 ± 13 ^ns^	83.44 ± 4.3 ^ns^	28.90 ± 1.2 ^ns^
	WT	103.5 ± 3.9	38.16 ± 1.4	1.70 ± 0.3	9.94 ± 0.9	20.75 ± 1.6	157 ± 11	84.52 ± 3.9	28.75 ± 1.2
	GN-3	105.2 ± 2.3 ^ns^	37.21 ± 1.3 ^ns^	1.62 ± 0.1 ^ns^	9.70 ± 0.4 ^ns^	20.99 ± 1.7 ^ns^	159 ± 10 ^ns^	84.30 ± 4.1 ^ns^	29.21 ± 1.5 ^ns^
T_2_	GN-5	104.8 ± 3.6 ^ns^	34.29 ± 1.9 ^ns^	1.56 ± 0.2 ^ns^	9.50 ± 0.6 ^ns^	20.26 ± 1.9 ^ns^	158 ± 12 ^ns^	85.80 ± 3.6 ^ns^	28.27 ± 1.2 ^ns^
	GN-9	108.6 ± 3.8 ^ns^	35.12 ± 1.3 ^ns^	1.59 ± 0.3 ^ns^	9.75 ± 0.5 ^ns^	21.60 ± 2.1 ^ns^	156 ± 13 ^ns^	83.83 ± 4.7 ^ns^	29.34 ± 1.3 ^ns^
	GN-13	103.3 ± 2.7 ^ns^	35.50 ± 1.6 ^ns^	1.54 ± 0.1 ^ns^	10.50 ± 0.7 ^ns^	21.40 ± 2.8 ^ns^	158 ± 12 ^ns^	82.85 ± 4.9 ^ns^	28.72 ± 1.4 ^ns^
	WT	107.5 ± 4.1	36.46 ± 1.3	1.58 ± 0.2	10.21 ± 0.8	21.15 ± 1.5	156 ± 13	83.12 ± 3.8	29.60 ± 1.4
	GN-3	101.4 ± 2.5 ^ns^	36.12 ± 1.6 ^ns^	1.69 ± 0.3 ^ns^	10.17 ± 0.3 ^ns^	20.41 ± 1.4 ^ns^	158 ± 11 ^ns^	83.65 ± 3.3 ^ns^	28.93 ± 1.3 ^ns^
T_3_	GN-5	105.9 ± 3.7 ^ns^	35.91 ± 1.5 ^ns^	1.62 ± 0.3 ^ns^	9.72 ± 0.5 ^ns^	20.55 ± 2.1 ^ns^	157 ± 12 ^ns^	85.00 ± 4.4 ^ns^	29.95 ± 1.2 ^ns^
	GN-9	104.6 ± 3.2 ^ns^	36.10 ± 1.4 ^ns^	1.67 ± 0.3 ^ns^	9.60 ± 0.6 ^ns^	20.95 ± 2.3 ^ns^	155 ± 14 ^ns^	85.52 ± 3.5 ^ns^	28.75 ± 1.5 ^ns^
	GN-13	106.7 ± 2.8 ^ns^	35.85 ± 1.7 ^ns^	1.60 ± 0.2 ^ns^	10.21 ± 0.7 ^ns^	21.75 ± 2.4 ^ns^	157 ± 12 ^ns^	84.28 ± 5.3 ^ns^	29.29 ± 1.2 ^ns^

WT (wild type); PH (plant height); FLL (flag leaf length); FLW (flag leaf width); PN (panicle number); PL (panicle length); GNPP (grain number per plant); SSR (seed setting rate); GWT (1000-grain weight). ^ns^ represents a non-significant difference. Student’s *t*-test, *p ≤* 0.01. The data is from the individual plant of T_0_ generation and the mean of five independent samples from T_1_, T_2_, and T_3_ generation.

**Table 3:** Important differentially expressed proteins related to photosynthesis, response to stimulus biotic, and abiotic stress.

Protein ID	Gene	Annotation	Regulation
Q6Z9C3	*Os08g0547100*	Probable 6-phosphogluconolactonase 3, chloroplastic	Up
Q5KQI6	*SNAT1*	Serotonin N-acetyltransferase 1, chloroplastic	Up
Q69PS6	*OsNTRA*	Thioredoxin reductase NTRA	Up
Q0IZQ2	*Os09g0556500*	Cysteine—tRNA ligase CPS1 homolog, chloroplastic	Up
Q6ATB2	*CRSH2*	Probable GTP diphosphokinase CRSH2, chloroplastic	Up
Q7XTK3	*AMI1*	Amidase 1	Up
Q6K8R2	*Cht6*	Pathogenesis related (PR)-3 chitinase 6	Up
Q67WM9	*OS9*	Protein OS-9 homolog	Up
Q75T45	*RSOsPR10*	Os12g0555000 protein	Up
Q10HD0	*RCABP89*	Chlorophyll a-b binding protein, chloroplastic	Up
Q6ESR4	*DHN1*	Dehydration stress-inducible protein 1	Up
Q6K4P5	*Os02g0787800*	diacylglycerol kinase	Up
Q84ZE8	*OsJ_24279*	Auxin-regulated protein-like	Up
A0A0P0XMI4	*Os09g0446200*	γ-tubulin complex component	Up
Q2R351	*LOC_Os11g33120*	Respiratory burst oxidase protein D	Up
A3BUY8	*OsJ_27981*	PEROXIDASE_4 domain-containing protein	Up
A0A0P0VAH1	*Os01g0849000*	Os01g0849000 protein	Up
Q75IS1	*Os05g0162000*	Peroxidase	Up
Q5U1N1	*prx62*	Peroxidase	Up
Q8HCQ0	*nad3*	NADH-ubiquinone oxidoreductase chain 3	Up

## Supplementary Materials

The following are available online at https://www.mdpi.com/2073-4425/11/7/735/s1, Table S1: All the primers used in the study, Table S2: Efficiency score and positions of both targets, Table S3: Detection of mutations on the putative off-target, Table S4: List of primers for off-targets, Table S5: Type of mutations in T0 generation obtained by two CRISPR/Cas9 constructs, Table S6: Segregation of CRISPR/Cas9-induced mutations in homozygous transgenic plants during the T0 to T1 generation, Figure S1: Structure of pYLCRISPR/Cas9Pubi-H binary vector with a fragment containing a modified ccdB flanking two BsaI sites, Figure S2: Embedded view of plasmids (a) pYLsgRNA-OsU6a (3289 bp); (b) cloning strategy of PYLsgRNA-OsU6a showing sticky ends created by cutting with two enzymes (BamH1 and HindIII), selected fragments replaced indicated in white and the remaining vector fragment is in black; (c) pYLsgRNA-OsU6b (3175 bp); (d) cloning strategy of PYLsgRNA-OsU6b showing sticky ends created by cutting with two enzymes (BamH1 and HindIII), selected fragments replaced indicated in white and the remaining vector fragment is in black, Figure S3: Vector map, schematic diagram, and target sites of sgRNAs in Pi21. (A) Vector map of Cas9/gRNA. LB: Vector left border; Ubi: ubiquitin promoter; pCas9, Cas9 protein; gRNA: Guided RNA; rU6: Rice U6 promoter; 35S: CaMV 35S promoter; Hyg: Hygromycin; RB, Vector right border; (B) exons are represented as black boxes. T1 and T2 represent Target1 and Target2, respectively. Target1 was from 1073 bp to 1092 bp and Target2 was 1130–1149 bp in the second exon, Figure S4: Secondary structures of sgRNAs (A) sgRNA1 and (B) sgRNA2, used in the experiment, Figure S5: Schematic representations, detection of T-DNA integration, and Pi21 target sequences assembly in vector. (A) Schematic representation of Target1 and Target2 driven by U6 promoters on vector; (B) gel electrophoresis detection of expression cassettes for target sites, M: DNA marker DL2000; (C) expression cassettes verification after the transformation of DH5α; M: DNA marker DL2000; bacterial colonies amplified; 1144 bp; (D) schematic presentation of Pi21 target regions displaying the location and corresponding sequences of gRNA1 and gRNA2 are represented in green while the PAM is underlined. Locations of forward and reverse primers flanked by the target region are highlighted in yellow and indicated with arrows, respectively; (E) sequencing peak map of both target sites assembled in pYLCRISPR/Cas9 Pubi-H vector; (F) T0 positive mutant lines; M: DL2000 DNA marker; WT: wild type; 1-25: T0 transgenic lines; (G) screening of T-DNA free GN-5 mutant lines; M: DL2000 DNA marker; WT: wild type, Figure S6: Identification and analysis of the proteome of wild type (WT) and CRISPR/Cas9 mutants. (A) The deviation among the measured molecular weight of the peptide precursor and the theoretical molecular weight; (B) protein molecular weight distribution. The X-axis represents the molecular weight and Y-axis is the number of identified proteins, Figure S7: GO and KEGG pathway enrichment histogram for down-regulated proteins. (A) DEPs GO enrichment, X-axis indicating the classification and the name of the GO terms. Y-axis represents the enrichment rate. (B) DEPs KEGG pathway enrichment histogram, X-axis indicating the classification and the name of the KEGG pathways. Y-axis represents the enrichment rate.
