# Supplementary material for: Knockout of Pi21 by CRISPR/Cas9 and iTRAQ-Based Proteomic Analysis of Mutants Revealed New Insights into M. oryzae Resistance in Elite Rice Line
Source: Genes (Basel). 2020 Jul 2;11(7):735. doi: 10.3390/genes11070735 (PMC7396999; doi:10.3390/genes11070735)
Supplement: Supplementary file 1 [file genes-11-00735-s001.zip › Supplementary file 1.docx]

Knockout of *Pi21* by CRISPR/Cas9 and iTRAQ-Based Proteomic Analysis of Mutants Revealed New Insights into *Magnaporthe oryzae* Resistance in Elite Rice Line

Gul Nawaz ^1^, Babar Usman ^1^, Haowen Peng^1^, Neng Zhao ^1^, Ruizhi Yuan^1^, Yaoguang Liu ^2^ * and Rongbai Li ^1^ *

^1^ College of Agriculture State Key Laboratory for Conservation and Utilization of Subtropical Agro-Bioresources, Guangxi University, Nanning 530004, China

^2^ State Key Laboratory for Conservation and Utilization of Subtropical Agricultural Bioresources, South China Agricultural University, Guangzhou, 510642, China

***** Correspondence: ygliu@scau.edu.cn, Tel: +86-20-8528-1908; lirongbai@126.com, Tel: +86-136-0009-4135

**Table S1**: All the primers used in the study

| **Primer name** | **Primer sequence (5'-3')** |
| --- | --- |
| Target 1 | GAAGCCGCCGCCGTGCAAGCCGG |
| Target 2 | GCCGAAGCCGGAGTGCAAGCTGG |
| *Pi21* F:  R: | GGAAGTTCGACCCGGAGAAG |
|  | TTCCGAGGCAAGGAAAGGAG |
| U-F | CTCCGTTTTACCTGTGGAATCG |
| gR-R | CGGAGGAAAATTCCATCCAC |
| U6aT1pi21-1-R | GCTTGCACGGCGGCGGCTTCggcagccaagccagca |
| gRT1pi21-1-F | AAGCCGCCGCCGTGCAAGCgttttagagctagaaat |
| U6bT2pi21-2-R | GCTTGCACTCCGGCTTCGGCaacacaagcggcagc |
| gRT2pi21-2-F | CCGAAGCCGGAGTGCAAGCgttttagagctagaaat |
| Pps-GGL | TTCAGAGGTCTCTCTCGACTAGTATGGAATCGGCAGCAAAGG |
| Pgs-GG2 | AGCGTGGGTCTCGTCAGGGTCCATCCACTCCAAGCTC |
| Pps-GG2 | TTCAGAGGTCTCTCTGACACTGGAATCGGCAGCAAAGG |
| Pgs-GGR | AGCGTGGGTCTCGACCGACGCGTATCCATCCACTCCAAGCTC |
| *HPT F:*  *HPT R:* | GTGCTTGACATTGGGGAGTT  ATTTGTGTACGCCCGACAGT |
| *Actin-F* | GAGTATGATGAGTCGGGTCCAG |
| *Actin-R* | ACACCAACAATCCCAAACAGAG |
| *Cas9-F* | CTGACGCTAACCTCGACAAG |
| *Cas9-R* | CCGATCTAGTAACATAGATGACACC |
| *SP-L1* | GCGGTGTCATCTATGTTACTAG |
| *SP-R* | GCCTATACCAAGTTATTGCA |
| *Pi-F* | GGCAAGATCATCAAGGAGATCC |
| *Pi-R* | CTTGGGCTTCTCGCAGTGA |
| *OsMKP1-F* | GCTGAATGCTCGTTAATGGTAG |
| *OsMKP1-R* | CTGCCCATTTTCTGACCATAAG |
| *OsAPx3-F* | TCCCAAGATTACCTATGCTGAC |
| *OsAPx3-R* | ATAATCTATAGTTGGCCCACCG |
| *OsDHAR1-F* | TCATTGAGGAGAAGTACCCAAC |
| *OsDHAR1-R* | GAAATGTTCTGCCCGTTGATAA |
| *OsLIR1-F* | CCATGGAGAATTGGAGTCTTCT |
| *OsLIR1-R* | GCTAGACTAGCGTGCCTTATTA |
| *OsGLO3-F* | CTGGGAGCTTCAGGAGTATTTA |
| *OsGLO3-R* | AGCATCTCAAATGGACTAGCAT |
| *OsINO1-1-F* | GATCGAGTCGGACTACCAGTA |
| *OsINO1-1-R* | GTGAGTGAGCCATAGTAGTTGG |
| *OsCHT6-F* | CGCCAACCACGAGACCATAAA |
| *OsCHT6-R* | TGCTCGTCTCGTCGCAGTA |
| *OsCRSH2-F* | GGAGGACAAGGATGATGAGTAC |
| *OsCRSH2-R* | ATTTTGCAGCTCGTTTGTTCAG |
| *OsPAL1-F* | GACCCTGTATTTTCTTCGTTCG |
| *OsPAL1-R* | AGTAGCAATACTTTCACCCCAA |
| *OsPR1a-F* | GGCCAATCTCCCTACTGATTAA |
| *OsPR1a-R* | GCATAAACACGTAGCATAGCAT |
| *OsPR10a-F* | CAGTGGTCAGTAGAGTGATCAG |
| *OsPR10a-R* | GGGTTAAGCTTCATGGTGTAGA |
| *OsAOS2-F* | CAATACGTGTACTGGTCGAATG |
| *OsAOS2-R* | CTTATTGCATATGCGTAGGACG |
| *OsRAP2-F* | AGAGAAAACCAGAAAAACGCTG |
| *OsRAP2-R* | TGCTTTGGGGATGGAATATGTA |
| *Osg1-F* | CGCTCCTGCTTATGTGATTTAC |
| *Osg1-R* | TTGTGACTTGTGAGGCTAGTAG |

Note: ACTAGT and ACGCGT: *SpeI* and *MluI* restriction enzyme cutting sites

| **Target Name** | **Position** | **Strand** | **Off Target Score** | **GC (%)** | **Region** | **Pairing with SgRNA** |
| --- | --- | --- | --- | --- | --- | --- |
| Target1 | 1073 - 1092 | + | 0.348 | 75.0 | CDS | None |
| Target2 | 1130 - 1149 | + | 0.450 | 70.0 | CDS | None |

**Table S2**: Efficiency score and positions of both targets.

| **Target** |  | **Name of putative off-target site** | **Genomic location** | **Sequence of the putative off-target site** | **Gene Locus** | **No. of mismatching bases** | **No. of plants sequenced** | **No. of plants with mutations** |
| --- | --- | --- | --- | --- | --- | --- | --- | --- |
| **Pi21T1** |  | OT1 | Chr3: -6521030 | GATGCTGCAGCCGAGCAAGC**CGG** | LOC_Os03g12360 | 4 | 35 | 0 |
|  |  | OT2 | Chr2: +23486424 | GAAGGCCCTGCCGTACAAGC**CGG** | LOC_Os02g38840 | 4 | 35 | 0 |
|  |  | OT3 | Chr4: +1409786 | GAAGACGTCACTGTGCAAGC**TGG** | LOC_Os04g03310 | 4 | 35 | 0 |
|  |  | OT4 | Chr2: +1344930 | CGAGGCGCCGCCGAGCAAGC**CGG** | LOC_Os02g03330 | 4 | 35 | 0 |
|  |  | OT5 | Chr11: +10186417 | GCACCCGCCGCCGTGCAACC**CGG** | LOC_Os11g18110 | 3 | 35 | 0 |
| **Pi21T2** |  | OT6 | Chr1: +30407237 | GCCGGGGCTGGAGTGCAAGC**CGG** | LOC_Os01g52880 | 3 | 35 | 0 |
|  |  | OT7 | Chr12: -13663538 | GACGAGGGCGGAGAGCAAGC**CGG** | LOC_Os12g24040 | 4 | 35 | 0 |
|  |  | OT8 | Chr6: +30364307 | GACGAGGGCGGAGAGCAAGC**CGG** | LOC_Os06g50140 | 4 | 35 | 0 |
|  |  | OT9 | Chr10: +21944686 | GCTGATGCCGGAGTACAGGC**CGG** | LOC_Os10g40810 | 4 | 35 | 0 |
|  |  | OT10 | Chr6: -4301825 | GCTGGAGCCGGAGAGCATGC**CGG** | LOC_Os06g08640 | 4 | 35 | 0 |

**Table S3**: Detection of mutations on the putative off-target

Note: The protospacer adjacent motif (PAM) (NGG) is shown in green background

**Table S4**: List of primers for off-targets.

| **Primer name** | **Primer sequence (5'-3')** |
| --- | --- |
| POT1 | F: GTCGACGAGGTGAAGGGG  R: CTCGATGTTCCACGGGTTCA |
| POT2 | F: TAAGAGGGGATCAGCAGCAC  R: AGCTCCTGATAAACCGGCAT |
| POT3 | F: TGAAGTGCTTGAGAGGAGAGA  R: TTTTCAGTCCCAGCCCAGTC |
| POT4 | F: CCTCTCATTTCCGCCTCTCT  R: CACCGCCATTAGTGTAGTGC |
| POT5 | F: ACGCACAATCCAACAAGCTT  R: CAACCCGAGCAAGACGAAG |
| POT6 | F: CTCCGTCGCTGTGTTCGT  R: GTTCTGGGAGATGGTGAGCA |
| POT7 | F: CCAAGCCTAACCCAAACGTC  R: TGACAGGTCTCGCAGTTCAT |
| POT8 | F: ATCTTTGTTCTTGGCAGGCG  R: CCGTCAGAATCCGAGCAATG |
| POT9 | F: GTAGCAAGAGGTCGCGCG |
|  | R: GCGGATGATGAGGAGCTCC |
| POT10 | F: TGGACGCCATGAACCTGC |
|  | R: TACGTGAATCCGGAAACTCC |

**Table S5.** Type of mutations in T_0_ generation obtained by two CRISPR/Cas9 constructs.

| **Targets/sgRNA** | **Event ID** | **Sequence PAM** | **Zygosity** | **mutations** | **Copy#** | **T-DNA** |
| --- | --- | --- | --- | --- | --- | --- |
|  | WT | GCGAGAAGCCGCCGCCGTGCAAGCCGGAGGA |  |  |  |  |
|  | GN-2 | GCGAGAAGCCGCCGCCGTGCAAGCCGGAGGA | Hetero | WT | 2 | + |
|  |  | GCGAGAAGCCGCCGCCGT---AGCCGGAGGA |  | 3d |  |  |
|  | GN-3 | GCGAGAAGCCGCC---------GCCGGAGGA | Homo | 9d | 1 | + |
| Target 1 | GN-5 | GCGAGAAGCCGCCGCCG-------------- | Homo | 51d | 1 | + |
|  | GN-9 | GCGAGAAGCCGCCGCCGTGCAACCGCCGGAGGA | Homo | 2i | 2 | + |
|  | GN-13 | GCGAGAAGCCGCCGCCG----AGCCGGAGGA | Homo | 4d | 1 | + |
|  | GN-19 | GCGAGAAGCCGCCGCCGTGCA----------- | Bi | 23d | 4 | + |
|  |  | GCGAGAAGCCGCCG------------------ |  | 40d |  |  |
|  | GN-23 | GCGAGAAGCCGCCGCCGTGCA--CCGGAGGA | Bi | 2d | 3 | + |
|  |  | GCGAGAAGCCGC-------------------- |  | 50d |  |  |
|  | GN-24 | GCGAGAAGCCGCCGCCGTGCAA-CCGGAGGA | Homo | 1d | 1 | + |
|  | GN-25 | GCGAGAAGCCGCCGCCGTGCAAGCCGGAGGA | Chimeric | WT | 2 |  |
|  |  | GCGAGAAGCCGCCGCCGT---AGCCGGAGGA |  | 3d |  |  |
|  |  | GCGAGAAGCCGCCGCCGT--AAGCCGGAGGA |  | 2d |  |  |
|  |  |  |  |  |  |  |
|  |  |  |  |  |  |  |
|  | WT | CGCCGCCGAAGCCGGAGTGCAAGCTGGTGCC |  |  |  |  |
|  | GN-2 | CGCCGCCGAAGCCGGAGTGCA-GCTGGTGCC | Bi | 1d | 3 | + |
|  |  | CGCCGCCGAAGCCGGAGTG—AGCTGGTGCC |  | 2d |  |  |
|  | GN-3 | CGCCGCCGAAGCCGGA------GCTGGTGCC | Homo | 6d | 1 | + |
| Target 2 | GN-5 | CGCCGCCGAAGCCGGAGTGCAAGCTGGTGCC | WT | WT | 1 | + |
|  | GN-7 | CGCCGCCGAAGCCGGAGTGCAAGCCTGGTGCC | Homo | 1i | 1 | + |
|  | GN-9 | CGCCGCCGAAGCCGGAGTGC---CTGGTGCC | Homo | 3d | 1 | + |
|  | GN-13 | CGCCGCCGAAGCC-------AAGCTGGTGCC | Homo | 7d | 1 | + |
|  | GN-15 | CGCCGCCGAAGCCGGA-----AGCTGGTGCC | Bi | 5d | 3 | + |
|  |  | CGCCGCCGAAGCCGGAGTGC-AGCTGGTGCC |  | 1d |  |  |
|  | GN-18 | CGCCGCCGAAGCCGGAGTGCAAGCTGGTGCC | Chimeric | WT | 1 |  |
|  |  | CGCCGCCGAAGCCGG------AGCTGGTGCC |  | 6d |  |  |
|  |  | CGCCGCCGAAGCCGGAGTGC-AGCTGGTGCC |  | 1d |  |  |
|  | GN-21 | CGCCGCCGAAGCCGG--------CTGGTGCC | Homo | 8d/8d | 1 | + |
|  | GN-25 | CGCCGCCGAAGCCGGAGTGCAAGCTGGTGCC | Hetero | WT | 4 | + |
|  |  | CGCCGCCGAAGCCGGAGT--AAGCTGGTGCC |  | 2d |  |  |

d: deletion, i: insertion and WT: wild type, Homo: Homozygous, Hertero: Heterozygous, Bi: Biallelic, the numbers in front of the letters indicate the number of nucleotides affected, PAM: Protospacer adjacent motif highlighted in green, Copy#: T0 plants with each type of mutation. +: T-DNA positive

**Table S6.** Segregation of CRISPR/Cas9-induced mutations in homozygous transgenic plants during the T_0_ to T_1_ generation

|  | **T_0_** | |  | **T_1_** | | |
| --- | --- | --- | --- | --- | --- | --- |
| **Targets/sgRNA** | **Mutants** | **Zygosity** | **Type of mutations** | **No. of plants tested** | **Segregation ratio** | **T-DNA** |
|  | GN-3 | Homozygous | 9d/9d | 50 | 50(9d/9d) | 32+ :18− |
|  | GN-5 | Homozygous | 51d/51d | 50 | 50(51d/51d) | 33+ :17− |
| Target 1 | GN-9 | Homozygous | 2i/2i | 50 | 50(2i/2i) | 36+ :14− |
|  | GN-13 | Homozygous | 4d/4d | 50 | 50(4d/4d) | 29+ :21− |
|  | GN-2 | Heterozygous | WT/3d | 50 | 14WT:18(3d/3d) :18(WT/3d) | 39+ :11− |
|  | GN-19 | Biallelic | 23d/40d | 50 | 23(23d/40d):12(23d/23d):15(40d/40d) | 37+ :13− |
|  | GN-3 | Homozygous | 6d/6d | 50 | 50(6d/6d) | 32+ :18− |
|  | GN-5 | Homozygous | WT | 50 | 50(WT) | 33+ :17− |
| Target 2 | GN-9 | Homozygous | 3d/3d | 50 | 50(3d/3d) | 36+ :14− |
|  | GN-13 | Homozygous | 7d/7d | 50 | 50(7d/7d) | 29+ :21− |
|  | GN-15 | Biallelic | 5d/1d | 50 | 25(23d/40d):11(23d/23d):14(40d/40d) | 42+ :8− |
|  | GN-25 | Heterozygous | WT/2d | 50 | 15WT:12(2d/2d) :23(WT/2d) | 41+ :9− |

d: deletion, i: insertion and WT: wild type. The numbers in front of the letters indicate the number of nucleotides affected. Corresponding mutations in two alleles are distinguished by ‘/’. +; T-DNA positive; −; T-DNA-free


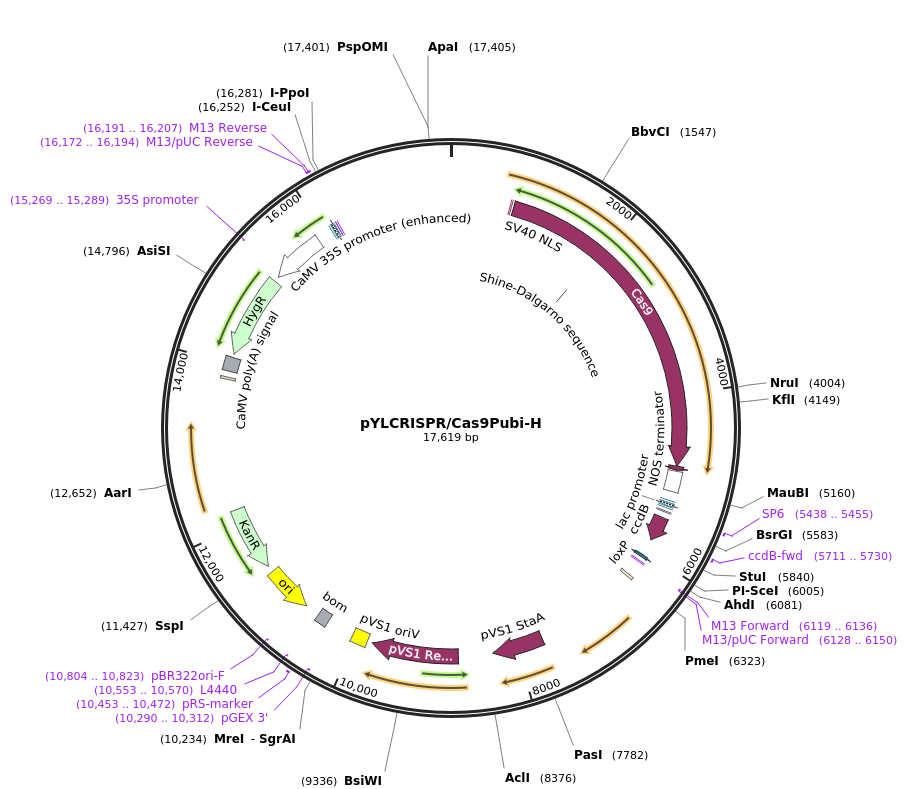
**Figure S1**: Structure of pYLCRISPR/Cas9Pubi-H binary vector with a fragment containing a modified ccdB flanking two BsaI sites.


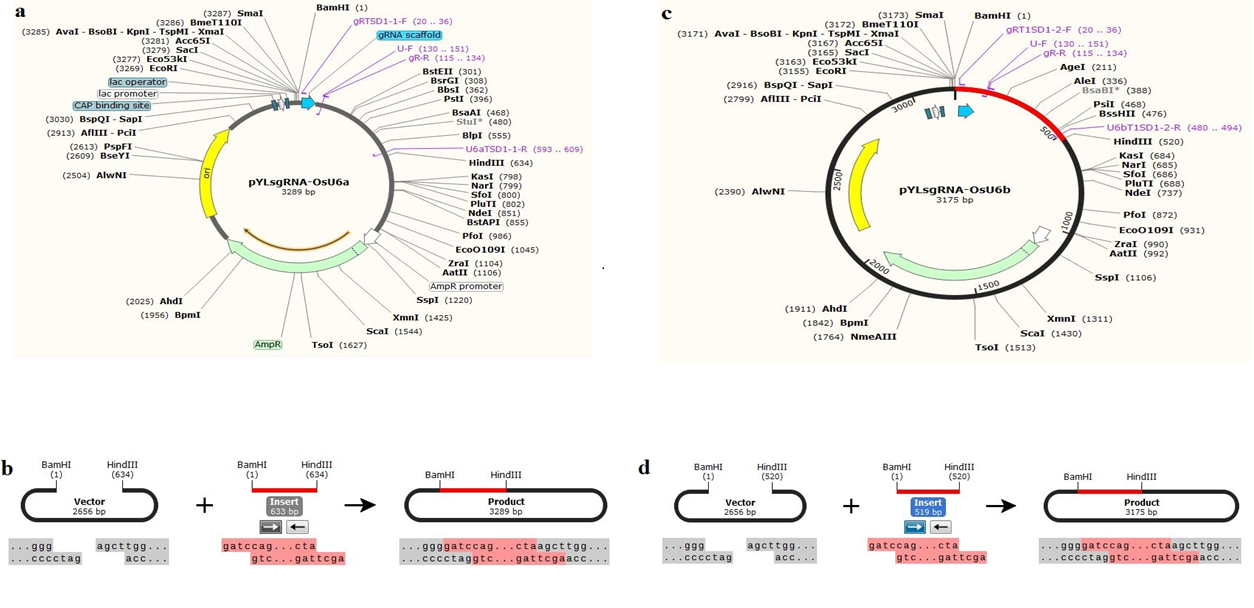


**Figure S2**: Embedded view of plasmids (a) pYLsgRNA-OsU6a (3289bp); (b) Cloning strategy of PYLsgRNA-OsU6a showing sticky ends created by cutting with two enzymes (BamH1 and HindIII), selected fragments replaced indicated in white and the remaining vector fragment is in black (c) pYLsgRNA-OsU6b (3175bp) (d) Cloning strategy of PYLsgRNA-OsU6b showing sticky ends created by cutting with two enzymes (BamH1 and HindIII), selected fragments replaced indicated in white and the remaining vector fragment is in black


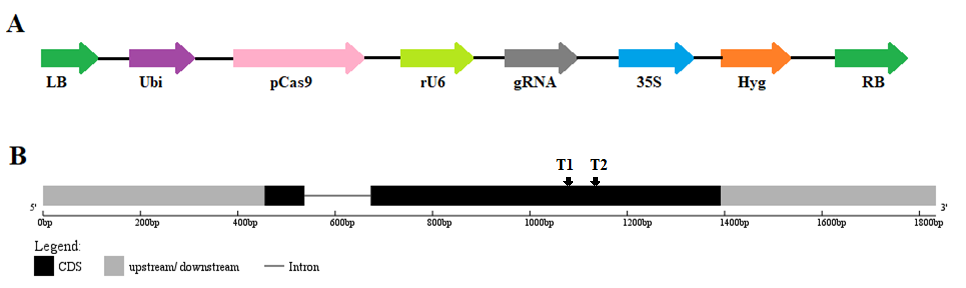


**Figure S3**: Vector map Schematic diagram and target sites of sgRNAs in Pi21. (A) Vector map of Cas9/gRNA. LB: Vector left border; Ubi: ubiquitin promoter; pCas9, Cas9 protein; gRNA: Guided RNA; rU6: Rice U6 promoter; 35S: CaMV 35S promoter; Hyg: Hygromycin; RB, Vector right border; (B) Exons are represented as black boxes. T_1_ and T_2_ represent Target1 and Target2, respectively. Target1 was from 1073bp – 1092bp and Target2 was 1130bp – 1149bp in the second exon


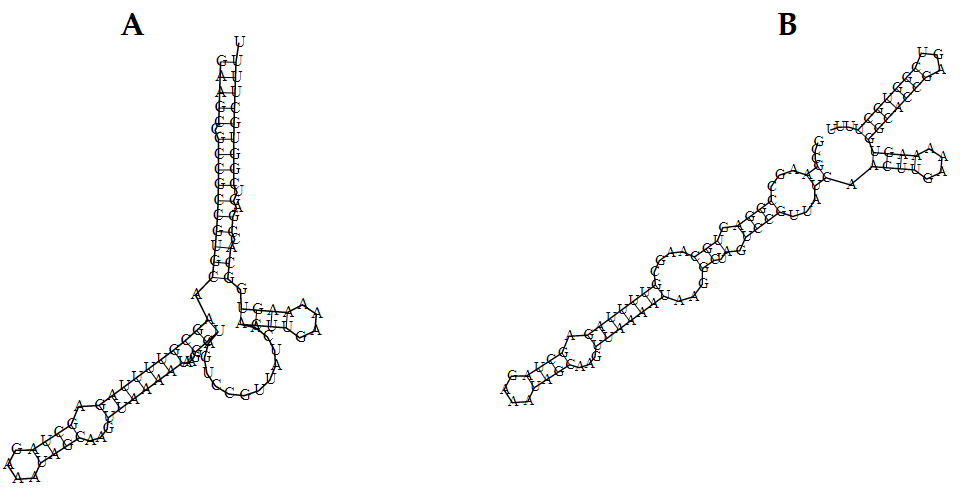


**Figure S4**: Secondary structures of sgRNAs (A) sgRNA1 and (B) sgRNA2, used in the experiment.


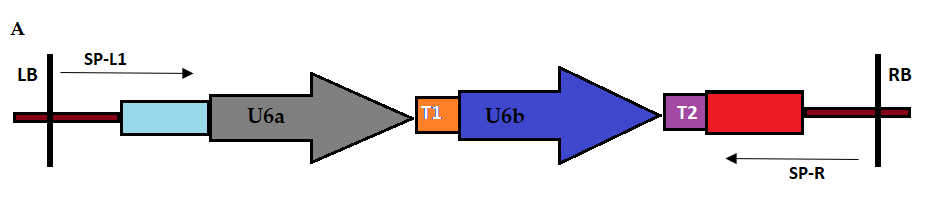


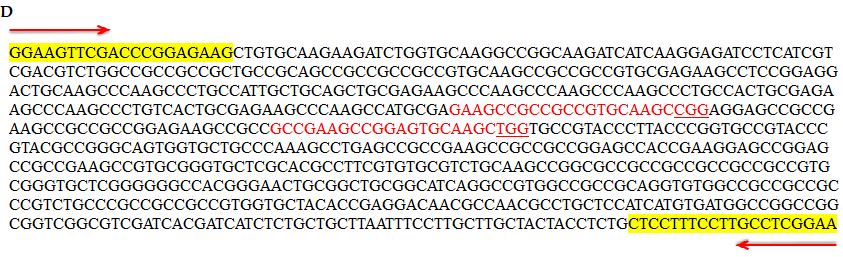

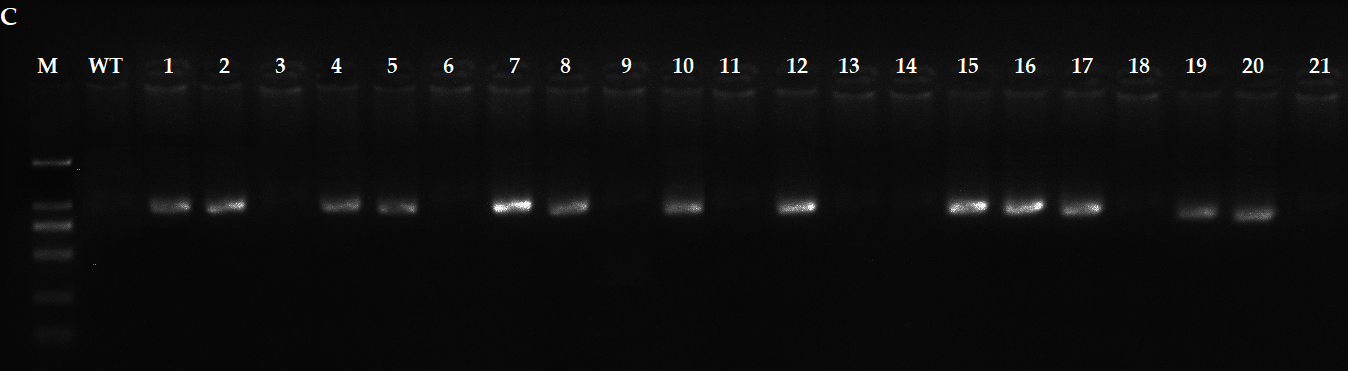

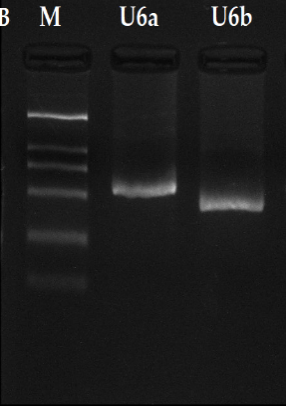


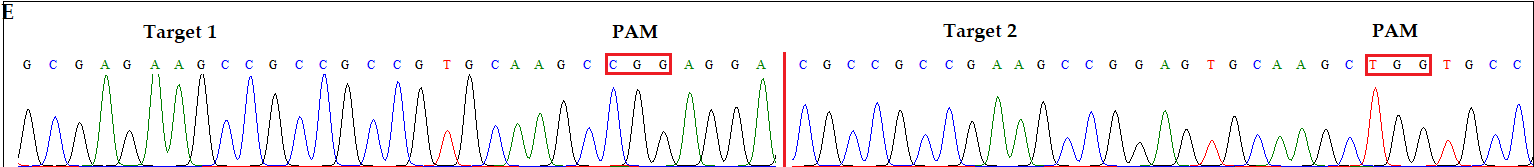


**
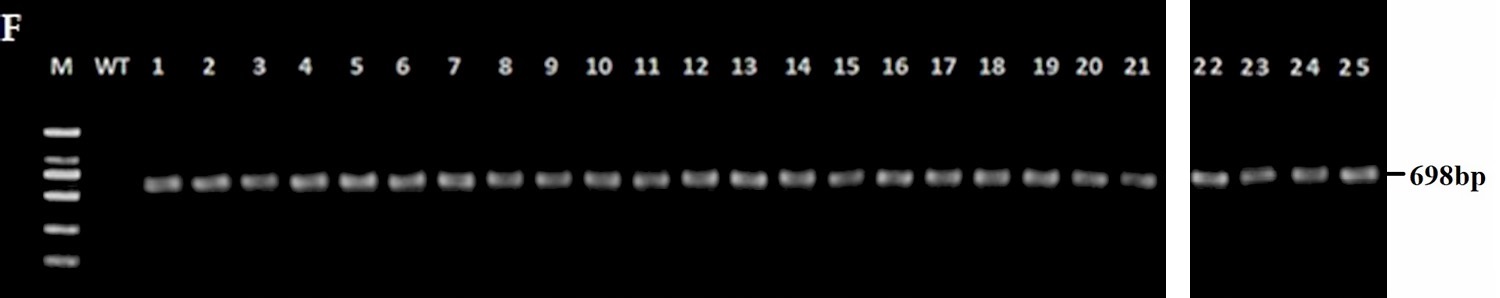
**

**
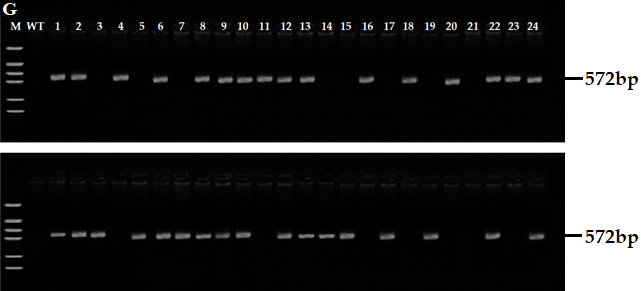
**

**Figure S5**: Schematic representations, detection of T-DNA integration and *Pi21* target sequences assembly in vector. (A) Schematic representation of Target1 and Target2 driven by U6 promoters on vector (B) Gel electrophoresis detection of expression cassettes for target sites, M: DNA marker DL2000; (C) Expression cassettes verification after the transformation of DH5α; M: DNA marker DL2000; bacterial colonies amplified; 1144 bp: (D) Schematic presentation of *Pi21* target regions displaying the location and corresponding sequences of gRNA1 and gRNA2 are represented in green while the PAM is underlined. Locations of forward and reverse primers flanked by the target region are highlighted in yellow and indicated with arrows, respectively; (E) Sequencing peak map of both target sites assembled in pYLCRISPR/Cas9 Pubi-H vector; (F) T0 positive mutant lines; M: DL2000 DNA marker; WT: wild type; 1-25: T0 transgenic lines (G) Screening of T-DNA free GN-5 mutant lines; M: DL2000 DNA marker; WT: wild type


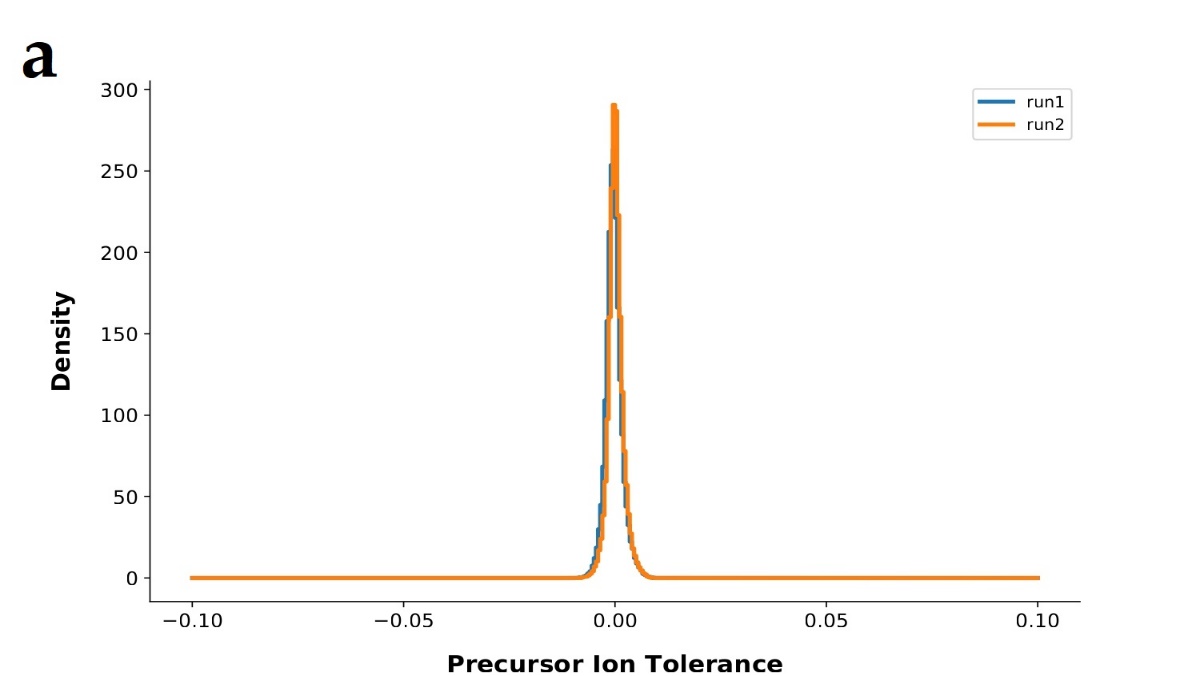


**
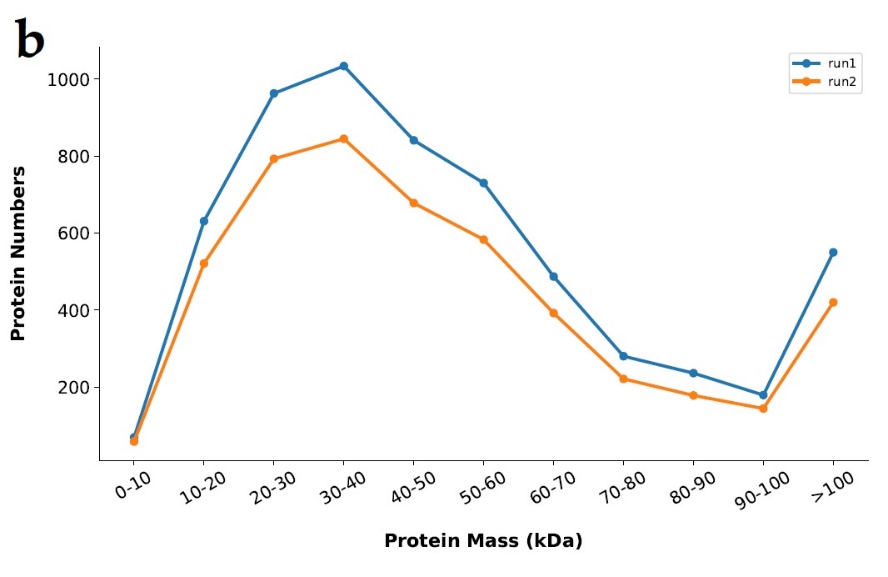
**

**Figure S6**: Identification and analysis of the proteome of wild type (WT) and CRISPR/Cas9 mutants. (**A**) The deviation amongst the measured molecular weight of the peptide precursor and the theoretical molecular weight; (**B**) Protein molecular weight distribution. The X-axis is representing molecular weight and Y-axis is the number of identified proteins


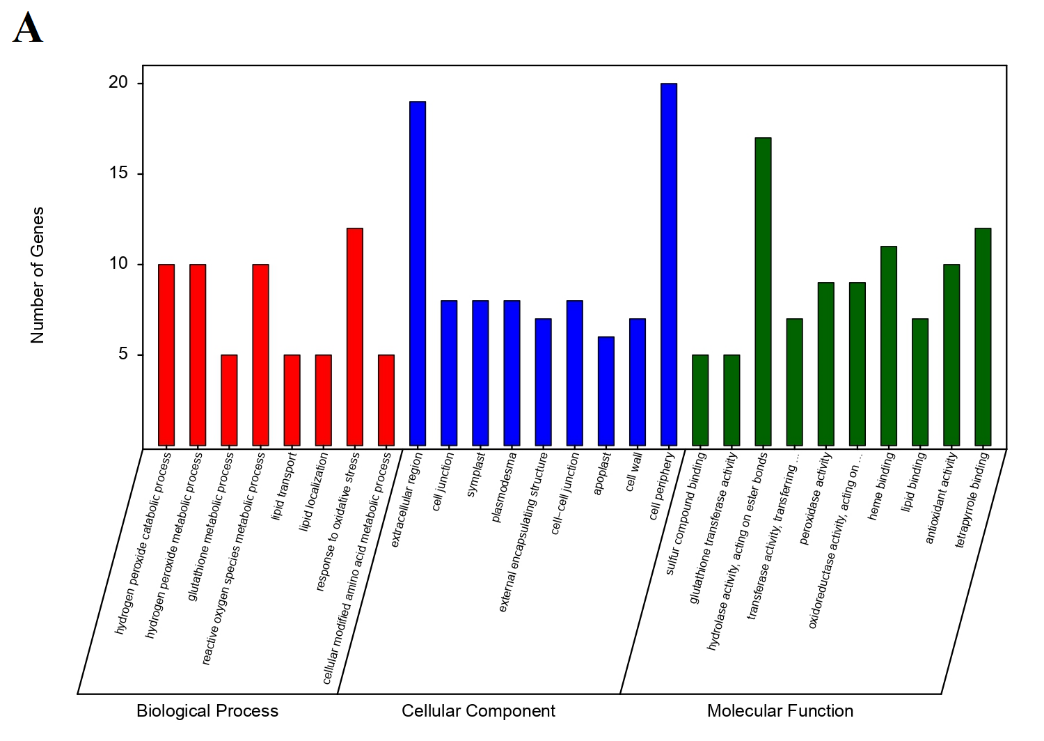


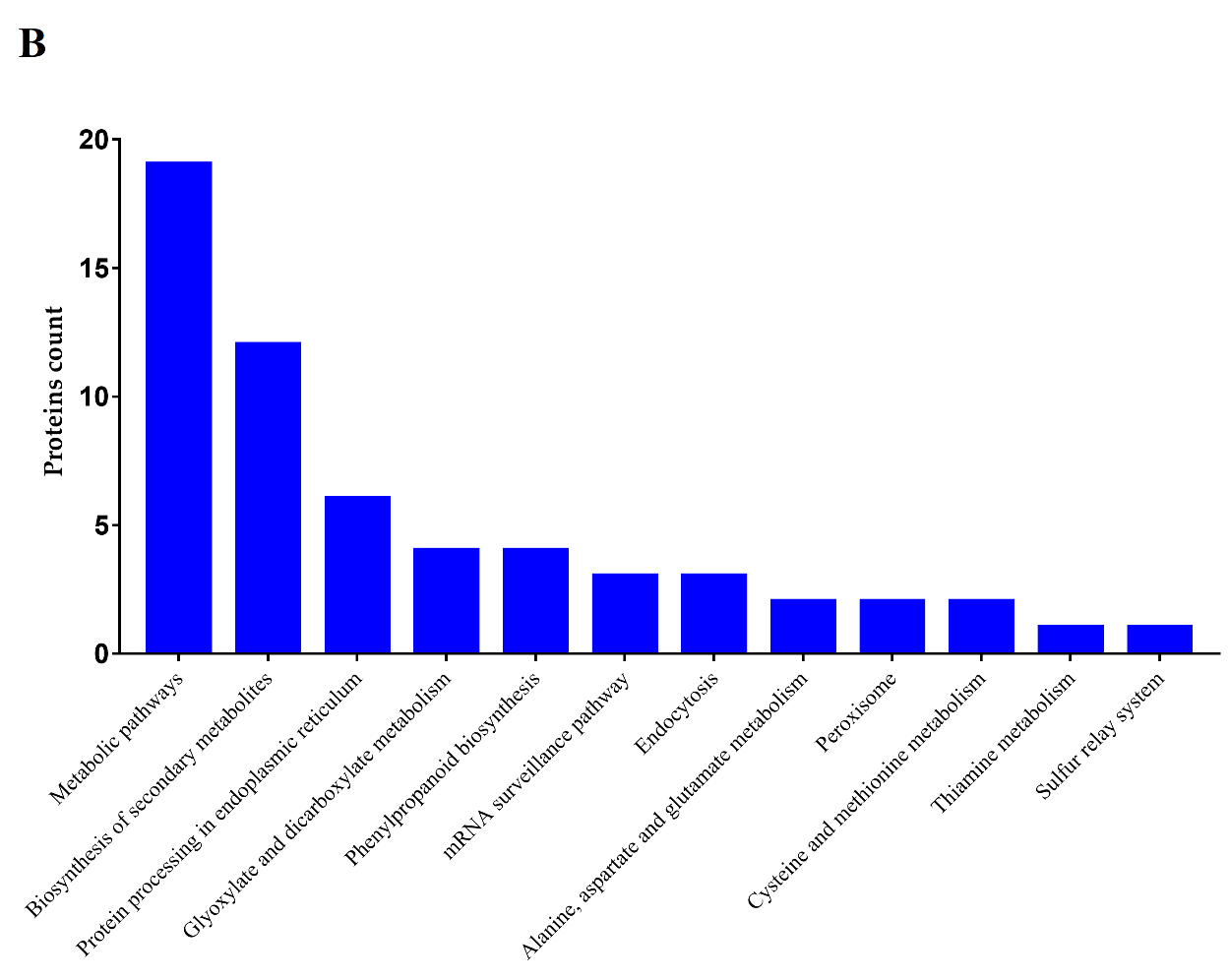


**Figure S7**: GO and KEGG pathway enrichment histogram for down-regulated proteins. (A) DEPs GO enrichment, X- axis indicating the classification and the name of the GO terms. Y-axis represents the enrichment rate. (B) DEPs KEGG pathway enrichment histogram, X-axis indicating the classification and the name of the KEGG pathways. Y-axis represents the enrichment rate
